# Di-μ-hydroxido-bis­[aqua­(pyridine-2,6-dicarboxyl­ato)iron(III)] monohydrate

**DOI:** 10.1107/S1600536810041966

**Published:** 2010-10-23

**Authors:** Hossein Eshtiagh-Hosseini, Nafiseh Alfi, Masoud Mirzaei, Philip Fanwick, Phillip E. Fanwick

**Affiliations:** a Department of Chemistry, School of Sciences, Ferdowsi University of Mashhad, Mashhad, Iran; b Department of Chemistry, Purdue University, W. Lafayette, IN 47907, USA

## Abstract

In the dinuclear title complex, [Fe_2_(OH)_2_(C_7_H_3_NO_4_)_2_(H_2_O)_2_]·H_2_O, the two Fe atoms are separated by 3.063 (1) Å. Inter­molecular O—H⋯O hydrogen bonds form an extensive three-dimensional hydrogen-bonding network, which consolidates the crystal packing.

## Related literature

The crystal structure of the anhydrous form of the title dinuclear complex has been reported by Thich *et al.* (1976 ▶). For related structures, see: Aghabozorg *et al.* (2008 ▶); Eshtiagh-Hosseini *et al.* (2010 ▶).
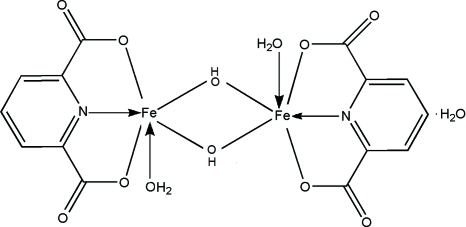


## Experimental

### 

#### Crystal data

[Fe_2_(OH)_2_(C_7_H_3_NO_4_)_2_(H_2_O_2_)_2_]·H_2_O
*M* *_r_* = 529.97
Monoclinic, 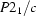

*a* = 11.4786 (11) Å
*b* = 21.7080 (16) Å
*c* = 7.3291 (6) Å
β = 90.099 (7)°
*V* = 1826.2 (3) Å^3^
*Z* = 4
Cu *K*α radiation
μ = 13.53 mm^−1^
*T* = 150 K
0.20 × 0.20 × 0.14 mm

#### Data collection

Rigaku Rapid II diffractometer
Absorption correction: multi-scan (*SCALEPACK*; Otwinowski & Minor, 1997 ▶) *T*_min_ = 0.120, *T*_max_ = 0.150
14275 measured reflections
2831 independent reflections
2695 reflections with *I* > 2σ(*I*)
*R*_int_ = 0.048

#### Refinement

*R*[*F* ^2^ > 2σ(*F* ^2^)] = 0.057
*wR*(*F* ^2^) = 0.153
*S* = 1.07
2831 reflections
311 parameters
H atoms treated by a mixture of independent and constrained refinement
Δρ_max_ = 0.97 e Å^−3^
Δρ_min_ = −0.76 e Å^−3^

### 

Data collection: *CrystalClear* (Rigaku, 2001 ▶); cell refinement: *DENZO*/*SCALEPACK* (Otwinowski & Minor, 1997 ▶); data reduction: *DENZO*/*SCALEPACK*; method used to solve structure: charge flipping (Oszlányi & Sütő, 2004 ▶) implemented in *PLATON* (Spek, 2009 ▶); program(s) used to refine structure: *SHELXL97* (Sheldrick, 2008 ▶); molecular graphics: *ORTEPII* (Johnson, 1976 ▶) and *PLATON*; software used to prepare material for publication: *SHELXL97*.

## Supplementary Material

Crystal structure: contains datablocks global, I. DOI: 10.1107/S1600536810041966/cv2763sup1.cif

Structure factors: contains datablocks I. DOI: 10.1107/S1600536810041966/cv2763Isup2.hkl

Additional supplementary materials:  crystallographic information; 3D view; checkCIF report

## Figures and Tables

**Table 1:** Hydrogen-bond geometry (Å, °)

*D*—H⋯*A*	*D*—H	H⋯*A*	*D*⋯*A*	*D*—H⋯*A*
O1—H1⋯O1W	0.70 (5)	2.34 (5)	2.966 (3)	151 (5)
O2—H2⋯O1W^i^	0.72 (5)	2.28 (5)	2.936 (3)	151 (5)
O15—H151⋯O12^ii^	0.83 (5)	1.78 (5)	2.611 (5)	173 (5)
O15—H152⋯O14^iii^	0.80 (4)	1.79 (4)	2.577 (4)	171 (5)
O25—H251⋯O24^iv^	0.78 (4)	1.80 (4)	2.577 (4)	176 (5)
O25—H252⋯O22^v^	0.71 (5)	1.87 (5)	2.576 (5)	173 (6)
O1W—H1W1⋯O23^iii^	0.87 (4)	2.10 (4)	2.962 (4)	176 (4)
O1W—H1W2⋯O13^iii^	0.87 (5)	2.22 (4)	3.072 (4)	169 (4)

Symmetry codes: (i) 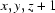; (ii) 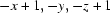; (iii) 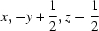; (iv) 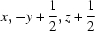; (v) 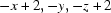.

**Fig. 1.**
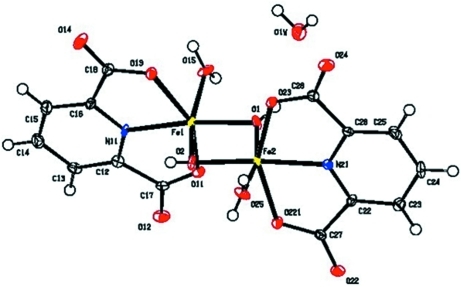
The molecular structure of I showing the atom-numbering scheme. Displacement ellipsoids are drawn at the 50% probability level.

## supplementary crystallographic information

### Comment

In continuation of our structural study of coordination compounds bearing different dicaroxylic acid and amine base fragments *via* proton transfer methodology (Aghabozorg *et al.*, 2008 and references therein; Eshtiagh-Hosseini *et al.* 2010), we present here the title compound, (I). The crystal structure of anhydrous form of I already has been reported by Thich *et al.* (1976); [(pydc)H_2_OFeOH]_2 _(II). The considerable feature of the title compound, is that the basic properties of (pydc)^2- ^did not allow 5-bromo-6-methyl-2-(4-methylpiperazine-1-yl)pyrimidine-4-amine (bmpa) participitate in the crystalline network. The other interesting point is that two Fe^III ^ions have different chemical environment making them non equivalent but with similar coordination geometry, that is, each Fe^III^ ion is hexa-coordinated by one tridentate (pydc)^2-^, two µ-hydroxo, and one coordinated water molecule (Fig. 1).

The above-mentioned complexes are very similar in terms of molecular structure but with different crystal structures. Both structures consist of [(pydc)H_2_OFe(OH)]_2_ dimeric units but the only difference between them is the peresence of a crystalline water molecule in the I.

In I (Fig. 1), all bond lengths and angles are normal and comparable with those observed in anhydrous form of the title dinuclear complex (Thich *et al.*, 1976). In the crystal structure, intermolecular O—H···O hydrogen bonds (Table 1) form an extensive three-dimensional hydrogen-bonding network, which consolidate the crystal packing. Indeed, firstly, covalent bonds cause to constructe dinuclear skeleton, and then the classical hydrogen bond interactions caused by O—H···O, lead to the self-aggregation process.

### Experimental

A solution of FeCl_3_.6H_2_O was added to a mixture of pydcH_2_ and bmpa in molar ratios of 1:2:2, respectively. The obtained solution was refluxed for 6 hrs in the 343 K. Suitable orange chunk [(µ-OH)_2_Fe_2_(pydc)_2_(H_2_O)_2_].H_2_O crystals for single crystal X-ray structure determination were obtained after slow evaporation of solvent in R.T.

### Refinement

C-bound H atoms were geometrically positioned (C—H 0.93 Å) and refined as riding, with U_iso_(H)=1.2U_eq_(C). O-bound H atoms were located on a difference map and isotropically refined.

### Crystal data

**Table:**

[Fe_2_(OH)_2_(C_7_H_3_NO_4_)_2_(H_2_O_2_)_2_]·H_2_O	*F*(000) = 1072
*M**_r_* = 529.97	*D*_x_ = 1.927 Mg m^−^^3^
Monoclinic, *P*2_1_/*c*	Cu - *K*α radiation, λ = 1.54184 Å
Hall symbol: -P 2ybc	Cell parameters from 2932 reflections
*a* = 11.4786 (11) Å	θ = 2–63°
*b* = 21.7080 (16) Å	µ = 13.53 mm^−^^1^
*c* = 7.3291 (6) Å	*T* = 150 K
β = 90.099 (7)°	Chunk, orange
*V* = 1826.2 (3) Å^3^	0.20 × 0.20 × 0.14 mm
*Z* = 4	

### Data collection

**Table:**

Rigaku Rapid II diffractometer	2695 reflections with *I* > 2σ(*I*)
confocal optics	*R*_int_ = 0.048
ω scans	θ_max_ = 63.3°, θ_min_ = 2.0°
Absorption correction: multi-scan (*SCALEPACK*; Otwinowski & Minor, 1997)	*h* = −11→12
*T*_min_ = 0.120, *T*_max_ = 0.150	*k* = −25→0
14275 measured reflections	*l* = 0→8
2831 independent reflections	

### Refinement

**Table:**

Refinement on *F*^2^	0 restraints
Least-squares matrix: full	H atoms treated by a mixture of independent and constrained refinement
*R*[*F*^2^ > 2σ(*F*^2^)] = 0.057	*w* = 1/[σ^2^(*F*_o_^2^) + (0.1304*P*)^2^ + 0.6186*P*] where *P* = (*F*_o_^2^ + 2*F*_c_^2^)/3
*wR*(*F*^2^) = 0.153	(Δ/σ)_max_ < 0.001
*S* = 1.07	Δρ_max_ = 0.97 e Å^−^^3^
2831 reflections	Δρ_min_ = −0.76 e Å^−^^3^
311 parameters	

### Special details

**Table:**

Geometry. All e.s.d.'s (except the e.s.d. in the dihedral angle between two l.s. planes) are estimated using the full covariance matrix. The cell e.s.d.'s are taken into account individually in the estimation of e.s.d.'s in distances, angles and torsion angles; correlations between e.s.d.'s in cell parameters are only used when they are defined by crystal symmetry. An approximate (isotropic) treatment of cell e.s.d.'s is used for estimating e.s.d.'s involving l.s. planes.
Refinement. Outlier data were removed using a local program based on the method of Prince and Nicholson [Prince, E. & Nicholson, W. L. (1983). *Acta Cryst.* A39, 407–410.]Refinement on *F*^2^ for ALL reflections except for 0 with very negative *F*^2^ or flagged by the user for potential systematic errors. Weighted *R*-factors *wR* and all goodnesses of fit *S* are based on *F*^2^, conventional *R*-factors *R* are based on *F*, with *F* set to zero for negative *F*^2^. The observed criterion of *F*^2^ > σ(*F*^2^) is used only for calculating R_factor_obs *etc*. and is not relevant to the choice of reflections for refinement. *R*-factors based on *F*^2^ are statistically about twice as large as those based on *F*, and *R*- factors based on ALL data will be even larger.

### Fractional atomic coordinates and isotropic or equivalent isotropic displacement parameters (Å^2^)

**Table:**

	*x*	*y*	*z*	*U*_iso_*/*U*_eq_	
Fe1	0.63100 (5)	0.12044 (2)	0.72067 (7)	0.0080 (3)	
Fe2	0.89320 (5)	0.11910 (2)	0.79919 (7)	0.0082 (3)	
O1	0.7813 (3)	0.11982 (10)	0.5976 (4)	0.0131 (7)	
O2	0.7419 (3)	0.11662 (10)	0.9247 (4)	0.0111 (6)	
O11	0.6011 (2)	0.02831 (10)	0.6877 (3)	0.0135 (6)	
O12	0.4819 (2)	−0.05113 (9)	0.7494 (3)	0.0161 (6)	
O13	0.57708 (19)	0.20747 (9)	0.8073 (3)	0.0115 (5)	
O14	0.4320 (2)	0.26115 (10)	0.9362 (3)	0.0186 (6)	
O15	0.5550 (2)	0.14072 (12)	0.4813 (3)	0.0159 (6)	
O1W	0.7628 (2)	0.19217 (14)	0.2554 (3)	0.0220 (7)	
O22	1.0531 (2)	−0.04764 (10)	0.7153 (3)	0.0163 (6)	
O23	0.9419 (2)	0.20979 (9)	0.7383 (3)	0.0133 (5)	
O24	1.0833 (2)	0.26950 (10)	0.6214 (3)	0.0191 (6)	
O25	0.9767 (2)	0.13003 (13)	1.0362 (4)	0.0150 (6)	
O221	0.9281 (2)	0.02666 (10)	0.8040 (3)	0.0118 (5)	
N11	0.4717 (2)	0.10535 (13)	0.8448 (4)	0.0076 (6)	
N21	1.0478 (3)	0.11096 (12)	0.6582 (4)	0.0091 (7)	
C12	0.4279 (3)	0.04863 (14)	0.8479 (4)	0.0109 (7)	
C13	0.3222 (3)	0.03599 (14)	0.9274 (4)	0.0123 (7)	
C14	0.2600 (3)	0.08500 (16)	1.0037 (4)	0.0164 (8)	
C15	0.3062 (3)	0.14464 (15)	0.9951 (4)	0.0154 (8)	
C16	0.4128 (3)	0.15301 (15)	0.9138 (4)	0.0111 (7)	
C17	0.5088 (3)	0.00355 (14)	0.7540 (4)	0.0110 (7)	
C18	0.4780 (3)	0.21304 (14)	0.8853 (4)	0.0116 (7)	
C22	1.0924 (3)	0.05521 (14)	0.6318 (4)	0.0100 (7)	
C23	1.1952 (3)	0.04648 (15)	0.5364 (4)	0.0131 (7)	
C24	1.2517 (3)	0.09854 (16)	0.4704 (4)	0.0156 (8)	
C25	1.2059 (3)	0.15709 (15)	0.5038 (4)	0.0149 (7)	
C26	1.1027 (3)	0.16140 (14)	0.5996 (4)	0.0100 (7)	
C27	1.0193 (3)	0.00617 (14)	0.7233 (4)	0.0110 (7)	
C28	1.0388 (3)	0.21935 (14)	0.6557 (4)	0.0125 (7)	
H1	0.799 (5)	0.132 (2)	0.514 (8)	0.026 (14)*	
H2	0.724 (5)	0.132 (2)	1.007 (8)	0.032 (15)*	
H13	0.2930	−0.0039	0.9304	0.015*	
H14	0.1886	0.0780	1.0597	0.020*	
H15	0.2656	0.1779	1.0435	0.019*	
H23	1.2253	0.0072	0.5172	0.016*	
H24	1.3201	0.0944	0.4037	0.019*	
H25	1.2440	0.1923	0.4627	0.018*	
H151	0.539 (4)	0.111 (2)	0.414 (7)	0.028 (12)*	
H152	0.522 (4)	0.173 (2)	0.474 (6)	0.041 (14)*	
H1W1	0.817 (4)	0.2200 (19)	0.255 (6)	0.025 (11)*	
H1W2	0.704 (4)	0.217 (2)	0.262 (6)	0.042 (15)*	
H251	1.006 (4)	0.161 (2)	1.063 (6)	0.039 (14)*	
H252	0.973 (5)	0.106 (2)	1.101 (8)	0.047*	

### Atomic displacement parameters (Å^2^)

**Table:**

	*U*^11^	*U*^22^	*U*^33^	*U*^12^	*U*^13^	*U*^23^
Fe1	0.0075 (4)	0.0056 (4)	0.0109 (4)	0.00088 (17)	0.0042 (2)	−0.00093 (16)
Fe2	0.0078 (4)	0.0058 (4)	0.0108 (4)	−0.00005 (17)	0.0047 (2)	0.00054 (16)
O1	0.0074 (17)	0.0223 (15)	0.0096 (14)	−0.0017 (9)	0.0030 (11)	0.0039 (9)
O2	0.0066 (16)	0.0154 (14)	0.0114 (14)	0.0015 (9)	0.0032 (11)	−0.0035 (9)
O11	0.0179 (15)	0.0068 (11)	0.0158 (12)	0.0024 (9)	0.0035 (10)	−0.0009 (8)
O12	0.0242 (15)	0.0072 (12)	0.0170 (11)	−0.0010 (9)	−0.0031 (10)	−0.0016 (8)
O13	0.0120 (14)	0.0062 (11)	0.0164 (11)	−0.0011 (9)	0.0047 (9)	−0.0019 (8)
O14	0.0146 (14)	0.0089 (11)	0.0323 (14)	0.0016 (9)	0.0049 (10)	−0.0058 (9)
O15	0.0231 (16)	0.0076 (13)	0.0169 (13)	0.0073 (11)	−0.0031 (10)	−0.0043 (10)
O1W	0.0215 (18)	0.0158 (15)	0.0288 (16)	−0.0023 (11)	0.0084 (12)	−0.0012 (9)
O22	0.0270 (16)	0.0077 (12)	0.0141 (11)	0.0037 (9)	0.0041 (10)	0.0003 (8)
O23	0.0142 (14)	0.0062 (11)	0.0195 (12)	0.0031 (9)	0.0070 (10)	0.0022 (8)
O24	0.0188 (15)	0.0076 (11)	0.0309 (13)	−0.0019 (10)	0.0039 (10)	0.0052 (10)
O25	0.0210 (16)	0.0102 (12)	0.0138 (13)	−0.0068 (11)	0.0003 (11)	0.0020 (9)
O221	0.0129 (14)	0.0061 (11)	0.0164 (11)	−0.0010 (9)	0.0046 (9)	−0.0012 (8)
N11	0.0020 (16)	0.0097 (13)	0.0111 (14)	0.0001 (11)	0.0014 (11)	−0.0024 (11)
N21	0.0082 (18)	0.0089 (14)	0.0103 (14)	−0.0002 (11)	0.0013 (12)	0.0023 (10)
C12	0.0107 (19)	0.0116 (16)	0.0104 (15)	−0.0015 (13)	−0.0023 (13)	−0.0011 (12)
C13	0.0108 (19)	0.0107 (15)	0.0155 (15)	−0.0052 (13)	−0.0020 (13)	0.0038 (12)
C14	0.011 (2)	0.0233 (18)	0.0154 (16)	−0.0001 (14)	0.0020 (13)	0.0030 (13)
C15	0.016 (2)	0.0147 (17)	0.0157 (16)	0.0010 (14)	0.0053 (14)	−0.0016 (13)
C16	0.012 (2)	0.0105 (16)	0.0104 (15)	0.0042 (13)	0.0022 (13)	−0.0016 (12)
C17	0.0147 (19)	0.0079 (16)	0.0106 (13)	0.0013 (13)	−0.0023 (13)	0.0018 (12)
C18	0.011 (2)	0.0104 (16)	0.0135 (15)	−0.0002 (13)	0.0005 (13)	−0.0014 (12)
C22	0.010 (2)	0.0086 (15)	0.0114 (15)	0.0008 (13)	0.0015 (12)	−0.0025 (12)
C23	0.014 (2)	0.0128 (16)	0.0123 (16)	0.0013 (13)	−0.0007 (13)	−0.0026 (12)
C24	0.010 (2)	0.0244 (18)	0.0128 (15)	−0.0009 (15)	0.0055 (13)	−0.0027 (14)
C25	0.015 (2)	0.0134 (16)	0.0163 (16)	−0.0044 (14)	0.0041 (14)	0.0026 (13)
C26	0.0128 (19)	0.0079 (15)	0.0093 (14)	0.0006 (13)	0.0013 (13)	0.0021 (12)
C27	0.015 (2)	0.0106 (16)	0.0078 (15)	0.0011 (13)	0.0004 (12)	0.0014 (12)
C28	0.015 (2)	0.0090 (16)	0.0141 (15)	−0.0010 (13)	0.0028 (14)	0.0011 (12)

### Geometric parameters (Å, °)

**Table:**

Fe1—O1	1.948 (3)	O25—H251	0.77 (5)
Fe1—O2	1.964 (3)	O25—H252	0.71 (5)
Fe1—O15	2.007 (2)	O221—C27	1.283 (4)
Fe1—O11	2.043 (2)	N11—C12	1.330 (4)
Fe1—N11	2.070 (3)	N11—C16	1.336 (4)
Fe1—O13	2.087 (2)	N21—C22	1.328 (4)
Fe2—O1	1.956 (3)	N21—C26	1.335 (4)
Fe2—O2	1.967 (3)	C12—C13	1.375 (5)
Fe2—O25	1.997 (3)	C12—C17	1.515 (4)
Fe2—O221	2.047 (2)	C13—C14	1.398 (5)
Fe2—N21	2.063 (3)	C13—H13	0.9300
Fe2—O23	2.095 (2)	C14—C15	1.400 (5)
O1—H1	0.70 (5)	C14—H14	0.9300
O2—H2	0.72 (5)	C15—C16	1.374 (5)
O11—C17	1.285 (4)	C15—H15	0.9300
O12—C17	1.227 (4)	C16—C18	1.517 (4)
O13—C18	1.280 (4)	C22—C23	1.385 (5)
O14—C18	1.229 (4)	C22—C27	1.513 (4)
O15—H151	0.82 (5)	C23—C24	1.390 (5)
O15—H152	0.80 (5)	C23—H23	0.9300
O1W—H1W1	0.87 (4)	C24—C25	1.397 (5)
O1W—H1W2	0.87 (5)	C24—H24	0.9300
O22—C27	1.232 (4)	C25—C26	1.382 (5)
O23—C28	1.284 (4)	C25—H25	0.9300
O24—C28	1.229 (4)	C26—C28	1.514 (4)
			
O1—Fe1—O2	77.23 (13)	C12—N11—Fe1	119.2 (2)
O1—Fe1—O15	88.89 (11)	C16—N11—Fe1	119.5 (2)
O2—Fe1—O15	162.77 (11)	C22—N21—C26	121.2 (3)
O1—Fe1—O11	94.99 (9)	C22—N21—Fe2	118.9 (2)
O2—Fe1—O11	99.04 (9)	C26—N21—Fe2	119.8 (2)
O15—Fe1—O11	92.22 (10)	N11—C12—C13	121.7 (3)
O1—Fe1—N11	170.43 (11)	N11—C12—C17	111.0 (3)
O2—Fe1—N11	103.34 (12)	C13—C12—C17	127.3 (3)
O15—Fe1—N11	92.06 (11)	C12—C13—C14	118.0 (3)
O11—Fe1—N11	75.46 (10)	C12—C13—H13	121.00
O1—Fe1—O13	114.22 (9)	C14—C13—H13	121.00
O2—Fe1—O13	89.93 (9)	C13—C14—C15	119.5 (3)
O15—Fe1—O13	86.48 (9)	C13—C14—H14	120.30
O11—Fe1—O13	150.70 (9)	C15—C14—H14	120.30
N11—Fe1—O13	75.34 (10)	C16—C15—C14	118.6 (3)
O1—Fe2—O2	76.95 (14)	C16—C15—H15	120.70
O1—Fe2—O25	165.85 (12)	C14—C15—H15	120.70
O2—Fe2—O25	91.08 (12)	N11—C16—C15	120.9 (3)
O1—Fe2—O221	98.57 (9)	N11—C16—C18	111.3 (3)
O2—Fe2—O221	97.93 (9)	C15—C16—C18	127.8 (3)
O25—Fe2—O221	90.46 (10)	O12—C17—O11	127.0 (3)
O1—Fe2—N21	100.77 (12)	O12—C17—C12	118.9 (3)
O2—Fe2—N21	173.12 (11)	O11—C17—C12	114.1 (3)
O25—Fe2—N21	91.96 (11)	O14—C18—O13	126.8 (3)
O221—Fe2—N21	75.88 (10)	O14—C18—C16	118.4 (3)
O1—Fe2—O23	90.39 (10)	O13—C18—C16	114.8 (3)
O2—Fe2—O23	111.18 (9)	N21—C22—C23	121.8 (3)
O25—Fe2—O23	86.90 (10)	N21—C22—C27	111.2 (3)
O221—Fe2—O23	150.80 (10)	C23—C22—C27	126.9 (3)
N21—Fe2—O23	75.16 (10)	C22—C23—C24	117.5 (3)
Fe1—O1—Fe2	103.38 (14)	C22—C23—H23	121.20
Fe1—O1—H1	132 (4)	C24—C23—H23	121.20
Fe2—O1—H1	118 (4)	C23—C24—C25	120.2 (3)
Fe1—O2—Fe2	102.37 (13)	C23—C24—H24	119.90
Fe1—O2—H2	116 (4)	C25—C24—H24	119.90
Fe2—O2—H2	129 (4)	C26—C25—C24	118.3 (3)
C17—O11—Fe1	120.21 (19)	C26—C25—H25	120.90
C18—O13—Fe1	119.0 (2)	C24—C25—H25	120.90
Fe1—O15—H151	117 (3)	N21—C26—C25	120.9 (3)
Fe1—O15—H152	117 (3)	N21—C26—C28	111.4 (3)
H151—O15—H152	123 (5)	C25—C26—C28	127.7 (3)
H1W1—O1W—H1W2	97 (5)	O22—C27—O221	127.4 (3)
C28—O23—Fe2	118.9 (2)	O22—C27—C22	118.1 (3)
Fe2—O25—H251	122 (3)	O221—C27—C22	114.5 (3)
Fe2—O25—H252	118 (5)	O24—C28—O23	126.9 (3)
H251—O25—H252	120 (6)	O24—C28—C26	118.6 (3)
C27—O221—Fe2	119.46 (19)	O23—C28—C26	114.5 (3)
C12—N11—C16	121.3 (3)		
			
O2—Fe1—O1—Fe2	−2.02 (11)	O2—Fe2—N21—C26	157.8 (8)
O15—Fe1—O1—Fe2	167.69 (12)	O25—Fe2—N21—C26	−86.0 (3)
O11—Fe1—O1—Fe2	−100.17 (11)	O221—Fe2—N21—C26	−176.0 (3)
N11—Fe1—O1—Fe2	−96.5 (6)	O23—Fe2—N21—C26	0.3 (2)
O13—Fe1—O1—Fe2	82.06 (12)	C16—N11—C12—C13	−2.2 (5)
O2—Fe2—O1—Fe1	2.02 (11)	Fe1—N11—C12—C13	−179.6 (2)
O25—Fe2—O1—Fe1	−30.9 (5)	C16—N11—C12—C17	177.4 (3)
O221—Fe2—O1—Fe1	98.23 (11)	Fe1—N11—C12—C17	0.1 (3)
N21—Fe2—O1—Fe1	175.38 (10)	N11—C12—C13—C14	0.9 (5)
O23—Fe2—O1—Fe1	−109.66 (10)	C17—C12—C13—C14	−178.7 (3)
O1—Fe1—O2—Fe2	2.00 (11)	C12—C13—C14—C15	0.6 (5)
O15—Fe1—O2—Fe2	−35.1 (4)	C13—C14—C15—C16	−1.0 (5)
O11—Fe1—O2—Fe2	95.11 (10)	C12—N11—C16—C15	1.9 (5)
N11—Fe1—O2—Fe2	172.19 (10)	Fe1—N11—C16—C15	179.2 (2)
O13—Fe1—O2—Fe2	−112.90 (10)	C12—N11—C16—C18	−177.1 (3)
O1—Fe2—O2—Fe1	−1.99 (11)	Fe1—N11—C16—C18	0.3 (3)
O25—Fe2—O2—Fe1	170.38 (11)	C14—C15—C16—N11	−0.2 (5)
O221—Fe2—O2—Fe1	−99.01 (10)	C14—C15—C16—C18	178.5 (3)
N21—Fe2—O2—Fe1	−73.4 (9)	Fe1—O11—C17—O12	−177.6 (2)
O23—Fe2—O2—Fe1	83.29 (12)	Fe1—O11—C17—C12	1.9 (3)
O1—Fe1—O11—C17	177.9 (2)	N11—C12—C17—O12	178.3 (3)
O2—Fe1—O11—C17	100.1 (2)	C13—C12—C17—O12	−2.1 (5)
O15—Fe1—O11—C17	−93.0 (2)	N11—C12—C17—O11	−1.2 (4)
N11—Fe1—O11—C17	−1.5 (2)	C13—C12—C17—O11	178.4 (3)
O13—Fe1—O11—C17	−6.3 (3)	Fe1—O13—C18—O14	−176.5 (3)
O1—Fe1—O13—C18	178.2 (2)	Fe1—O13—C18—C16	2.9 (3)
O2—Fe1—O13—C18	−105.9 (2)	N11—C16—C18—O14	177.4 (3)
O15—Fe1—O13—C18	91.0 (2)	C15—C16—C18—O14	−1.4 (5)
O11—Fe1—O13—C18	2.7 (3)	N11—C16—C18—O13	−2.0 (4)
N11—Fe1—O13—C18	−2.1 (2)	C15—C16—C18—O13	179.1 (3)
O1—Fe2—O23—C28	−103.7 (2)	C26—N21—C22—C23	−2.6 (5)
O2—Fe2—O23—C28	−179.9 (2)	Fe2—N21—C22—C23	179.2 (2)
O25—Fe2—O23—C28	90.2 (2)	C26—N21—C22—C27	175.4 (3)
O221—Fe2—O23—C28	4.8 (3)	Fe2—N21—C22—C27	−2.7 (4)
N21—Fe2—O23—C28	−2.7 (2)	N21—C22—C23—C24	0.8 (5)
O1—Fe2—O221—C27	97.8 (2)	C27—C22—C23—C24	−176.9 (3)
O2—Fe2—O221—C27	175.7 (2)	C22—C23—C24—C25	1.2 (5)
O25—Fe2—O221—C27	−93.1 (2)	C23—C24—C25—C26	−1.5 (5)
N21—Fe2—O221—C27	−1.2 (2)	C22—N21—C26—C25	2.3 (5)
O23—Fe2—O221—C27	−8.6 (3)	Fe2—N21—C26—C25	−179.5 (2)
O1—Fe1—N11—C12	−3.1 (8)	C22—N21—C26—C28	−176.5 (3)
O2—Fe1—N11—C12	−95.4 (2)	Fe2—N21—C26—C28	1.7 (4)
O15—Fe1—N11—C12	92.4 (2)	C24—C25—C26—N21	−0.2 (5)
O11—Fe1—N11—C12	0.7 (2)	C24—C25—C26—C28	178.3 (3)
O13—Fe1—N11—C12	178.2 (3)	Fe2—O221—C27—O22	179.1 (2)
O1—Fe1—N11—C16	179.5 (6)	Fe2—O221—C27—C22	0.1 (3)
O2—Fe1—N11—C16	87.2 (2)	N21—C22—C27—O22	−177.4 (3)
O15—Fe1—N11—C16	−85.0 (2)	C23—C22—C27—O22	0.5 (5)
O11—Fe1—N11—C16	−176.8 (3)	N21—C22—C27—O221	1.7 (4)
O13—Fe1—N11—C16	0.8 (2)	C23—C22—C27—O221	179.6 (3)
O1—Fe2—N21—C22	−94.0 (2)	Fe2—O23—C28—O24	−174.8 (3)
O2—Fe2—N21—C22	−24.0 (10)	Fe2—O23—C28—C26	4.3 (3)
O25—Fe2—N21—C22	92.2 (3)	N21—C26—C28—O24	175.3 (3)
O221—Fe2—N21—C22	2.2 (2)	C25—C26—C28—O24	−3.4 (5)
O23—Fe2—N21—C22	178.5 (3)	N21—C26—C28—O23	−3.8 (4)
O1—Fe2—N21—C26	87.8 (3)	C25—C26—C28—O23	177.5 (3)

### Hydrogen-bond geometry (Å, °)

**Table:**

*D*—H···*A*	*D*—H	H···*A*	*D*···*A*	*D*—H···*A*
O1—H1···O1W	0.70 (5)	2.34 (5)	2.966 (3)	151 (5)
O2—H2···O1W^i^	0.72 (5)	2.28 (5)	2.936 (3)	151 (5)
O15—H151···O12^ii^	0.83 (5)	1.78 (5)	2.611 (5)	173 (5)
O15—H152···O14^iii^	0.80 (4)	1.79 (4)	2.577 (4)	171 (5)
O25—H251···O24^iv^	0.78 (4)	1.80 (4)	2.577 (4)	176 (5)
O25—H252···O22^v^	0.71 (5)	1.87 (5)	2.576 (5)	173 (6)
O1W—H1W1···O23^iii^	0.87 (4)	2.10 (4)	2.962 (4)	176 (4)
O1W—H1W2···O13^iii^	0.87 (5)	2.22 (4)	3.072 (4)	169 (4)

Symmetry codes: (i) *x*, *y*, *z*+1; (ii) −*x*+1, −*y*, −*z*+1; (iii) *x*, −*y*+1/2, *z*−1/2; (iv) *x*, −*y*+1/2, *z*+1/2; (v) −*x*+2, −*y*, −*z*+2.
